# Outcomes of second opinions in general internal medicine

**DOI:** 10.1371/journal.pone.0236048

**Published:** 2020-07-09

**Authors:** Pascal M. Burger, Jan Westerink, Bram E. L. Vrijsen

**Affiliations:** 1 Department of Internal Medicine, University Medical Center Utrecht, Utrecht, The Netherlands; 2 Department of Vascular Medicine, University Medical Center Utrecht, Utrecht, The Netherlands; 3 Department of Acute Internal Medicine, University Medical Center Utrecht, Utrecht, The Netherlands; Chang Gung Memorial Hospital at Linkou, TAIWAN

## Abstract

**Background:**

To date, the outcomes of second opinions in internal medicine in terms of diagnostic yield and patient benefit have not been studied extensively. This retrospective study explores the outcomes of second opinions at a general internal medicine outpatient clinic in an academic hospital.

**Methods:**

A register of all patients referred to the general internal medicine outpatient clinic of the University Medical Center in Utrecht for a second opinion, was kept. All 173 patients referred between June 2016 and August 2018 were selected. Case records were analyzed for patient characteristics, referring doctor, chief complaint, performed investigations, follow-up time and, established diagnosis, additional diagnoses, initiated treatment and reported benefit.

**Results:**

A new diagnosis was established in 13% of all patients. A new treatment was initiated in 56% of all patients: 91% and 51% of patients with and without a new diagnosis respectively (p < 0.001). Of all patients, 19% received an effective treatment (52% vs 14% of patients with vs without a new diagnosis, p < 0.001). Regardless of treatment, resolution or improvement of the chief complaint was achieved in 28% of all patients (52% vs 25% of patients with vs without a new diagnosis, p = 0.006). Regarding diagnostics, 23-33% of radiology, endoscopy and pathology tests performed during second opinion were a repetition of previously conducted investigations. Conventional blood tests were a repetition in 89% of cases. Median time to diagnosis was 64 days (IQR: 25–128 days) and median time to discharge was 75 days (IQR: 31–144 days).

**Conclusion:**

Second opinions in general internal medicine lead to the establishment of a new diagnosis in a small proportion of patients. However, the value of second opinions may not be limited to the establishment of diagnoses, as new treatments are often initiated and overall patients report improved symptomatology in 28% of cases.

## Introduction

A second opinion is defined as a reevaluation of the diagnosis and/or treatment given by a doctor, carried out by a second, independent doctor from the same medical field [1, 2]. Patients request second opinions for various reasons [3–6]. Mostly, second opinions are requested when no explanation for the patients’ complaints is found by the original doctor, or when treatment is ineffective.

Over the years, many studies in various medical specialties and multiple countries have shown that second opinions lead to the establishment of a new diagnosis in 2-60% [7–30] and a change in treatment in 20-60% of patients [8–19, 21, 22, 24–27, 31]. In addition, studies have shown that patients are generally satisfied with the process, even if it has not led to a new diagnosis or treatment [7, 9, 12, 32]. Studies exploring the outcome of second opinions in general internal medicine have shown that a new diagnosis is established in only approximately 10% of patients [7, 8]. However, to date, only a limited number of observational studies have been carried out in this field. Moreover, previous studies did not evaluate the establishment of additional diagnoses, treatment initiation and effects, patient-reported symptomatology and relevance of performed investigations in the context of second opinions, in all patients [7, 8].

This raises the question as to what the actual outcomes of second opinions in internal medicine are, when studied extensively. Therefore, the aim of this study is to determine the outcomes of second opinions in a general internal medicine outpatient clinic in an academic hospital. Primarily, this study will assess in how many patients a new diagnosis was established during second opinion. Secondarily, this study will assess established additional diagnoses, initiated treatment and its effects, patient-reported symptomatology, relevance of (repeated) diagnostic investigations and time to diagnosis and time spent in the clinic during second opinion.

## Methods

### Second opinions in the Dutch health care system

In the Dutch health care system, for every medical issue, a patient’s initial consultation is always with a general practitioner. The general practitioner acts as a gatekeeper to hospital and specialist care. The general practitioner can decide to refer patients to a hospital for specialist care. This can be either a regular hospital or an academic hospital. After patients have received specialist care in a regular or academic hospital, they have a legal right to demand a second opinion, and second opinions are covered by basic insurance [2]. If patients demand a second opinion, they are then referred to another hospital by their original physician or their general practitioner. Again, this can be either a regular or an academic hospital. Physicians from regular and academic hospitals have similar levels of expertise, and have similar diagnostic resources at their disposal. So, second opinions are carried out on the same level of care as the first opinions.

### Study design

This study is a retrospective analysis of data retrieved from case records, stored in the electronic hospital information system at the University Medical Center in Utrecht (UMC Utrecht), an academic hospital in the Netherlands. Due to the retrospective and non-invasive nature of the study, it was not subject to the Dutch Medical Research Involving Human Subjects Act and formal consent was not required. The study was approved by the Medical Ethics Review Committee in the UMC Utrecht before data acquisition. Starting from June 2016, a register of all patients referred to the general internal medicine outpatient clinic of the UMC Utrecht for a second opinion, has been kept for administrative reasons. All patients referred for a second opinion between June 2016 and August 2018 were considered for this study, so there would be at least eight months between time of referral and the start of this study (1 May 2019). Patients who did not visit the clinic or visited the clinic of a different medical specialty were excluded. For all included patients, age at time of referral, gender and the following measures were collected from case records by the first researcher (PB).

A glossary of terms used throughout the manuscript is provided in S1 Table.

### Referral

Case records were screened for referring doctor, dates of last consultation with the previous physician and first consultation with the physician formulating the second opinion, and chief complaint. Referring doctor was based on the referral letter, and divided into three groups: general practitioner, locum general practitioner and medical specialist. Time between consultations was calculated. This was defined as the number of days between the last consultation with a previous physician (which was based on dates specified in the referral letter) and the first consultation with the physician formulating the second opinion. Chief complaint of the patient was based on the main complaint mentioned by the patient during the first visit to the clinic, as documented in the case record by the doctor formulating the second opinion. Chief complaints were divided into the following groups: fatigue, abdominal pain, pain (multifocal), weight loss, edema, fever, and other, based upon observed frequencies.

### Diagnosis

Case records were analyzed for diagnosis at time of referral, diagnosis by the doctor formulating the second opinion, diagnosis established during inter-collegial consultation (consultation by a doctor from another medical specialty, requested by the doctor formulating the second opinion) and additional diagnoses established in the context of the second opinion. Whether these types of diagnoses were established and the actual diagnoses were noted for the different types of diagnoses separately. Diagnoses were only included if they were considered definitive diagnoses, using the following definition: a diagnosis is said to be a definitive diagnosis when the treating physician concludes that the diagnosis has been established and that no further investigations to confirm this diagnosis are required. Diagnosis by referring doctor was based on the referral letter. If a diagnosis was established by the doctor formulating the second opinion, it was documented whether it was a new diagnosis: new diagnosis was defined as the establishment of a diagnosis different from the diagnosis at time of referral, or the establishment of a diagnosis in patients without a diagnosis at time of referral. The same was done for diagnoses established during inter-collegial consultation. Inter-collegial consultation was seen as a part of second opinions, and therefore, diagnoses established during inter-collegial consultation were added to diagnoses established by the doctors formulating the second opinions, when analyzing outcome of second opinions. Finally, for additional diagnoses established during second opinion, relevance was determined. An additional diagnosis was considered relevant only if it led to treatment for this diagnosis.

### Treatment and patient-reported symptomatology

Records were analyzed for treatment initiated by the doctor formulating the second opinion and changes made to pre-existing management plans, and their effects on the chief complaint. Treatment was divided into the following groups: newly prescribed medication, change in medication (dosage) used at time of referral, vitamin/iron supplementation (vitamin B11/B12/D and iron), analgesia (local anesthetics or transcutaneous electric nerve stimulation), physical therapy, cognitive behavioral therapy (CBT), change in diet, surgery (for example gastroenterological surgery) and other (for example radiotherapy). Treatment effects were based on patient opinion as documented by the doctor in the case record, and were divided into four groups: resolution, improvement, unchanged and worsened. An effective treatment was defined as a treatment leading to the resolution or improvement of the chief complaint. In a similar same way, we also analyzed case records of all patients for patient-reported symptomatology at the end of second opinions. The same four groups were used to define the outcome.

### Investigations

Investigations performed in the context of the second opinion were collected from case records: blood tests, urinalysis, microbiology tests, radiological tests, endoscopic procedures and pathology tests. Laboratory tests were divided into conventional blood tests (specified in S2 Table) and additional blood tests. Radiological tests were divided into X-ray, sonography, CT, MRI and PET/SPECT. Microbiology tests were divided into the following groups: viral, bacterial and other (parasites, fungi, protozoa). For every investigation was noted whether it was a new investigation or a repetition of a previous investigation. An investigation was considered a repeated investigation if the investigation had already been performed by a previous physician before the start of the second opinion and the exact same investigation was then performed again during the second opinion. If the results of previous investigations, or images or tissue obtained by radiology, endoscopy or pathology were transferred from a hospital of a previous physician to the UMC Utrecht, and were reassessed by a physician of the UMC Utrecht during the second opinion, this was not considered an investigation or a repeated investigation as the actual investigation was not performed during second opinion. For every investigation was also noted whether it led to any form of relevant information. Relevant information of any form, was defined as information not known from previous investigations leading to either the establishment of a diagnosis or additional diagnosis, the initiation of a new treatment or the requirement for another investigation for further assessment. Finally, for every investigation was noted whether it had shown anomalous results contributing to the establishment of a diagnosis.

### Follow-up

For each patient was noted whether the entire diagnostic process was completed, or the diagnostic process was still ongoing or the patient was lost to follow-up. Time to diagnosis, time to discharge from the clinic and time spent in the clinic were collected from case records. Time to diagnosis was defined as the number of days between the first visit to the clinic and the moment the diagnosis was established and discussed with the patient. Time to discharge from the clinic was defined as the number of days between the first visit to the clinic and the last visit to the clinic, or other departments of the hospital, as part of the diagnostic process or treatment of the chief complaint. If patients had not been discharged by the start of this study (1 May 2019), time to discharge from the clinic was defined as the number of days between the first visit to the clinic and 1 May 2019. For patients that were lost to follow-up, time to discharge from the clinic was defined as the number of days between the first and the last visit to the clinic (or other departments of the hospital), and was reported separately. Time spent in the clinic was defined as the total amount of time (in minutes) reserved for the patients’ appointments at the internal medicine outpatient clinic, as well as for appointments by phone. Total time spent in the clinic was calculated similarly, but all appointments regarding the chief complaint at any outpatient clinic in the UMC Utrecht were included.

### Validation of outcomes

After all data were collected from case records by the first researcher (PB), all established diagnoses and additional diagnoses were evaluated, also based on case record examinations, by two experienced internists (the two other authors: JW-BV). In three cases (1 diagnosis, 2 additional diagnoses) opinions differed between authors, and consensus was reached through group discussion involving all three authors (PB-JW-BV). In all other cases, authors agreed on the validity of the (additional) diagnoses collected from case records by the first researcher. Besides (additional) diagnoses, treatment including treatment effects and time to diagnosis were also checked by a second researcher (BV) in a random sample of 5% of all patients (N = 9). This was done to ensure that outcome definitions were adequately described, so that usage of the definitions by two independent researchers would lead to consistent results. All outcomes of patients from the sample determined by the second researcher were consistent with the outcomes determined by the first researcher.

### Statistical analyses

Descriptive statistics were used to summarize patients’ characteristics at baseline and established diagnoses, initiated treatment, follow-up times and performed investigations in the context of the second opinion. Categorical variables were characterized using frequencies and percentages, continuous variables were characterized using means and standard deviations or medians and interquartile ranges, when appropriate.

In order to compare outcome between groups of categorical/dichotomous variables, such as gender, referring doctor, chief complaint and groups of patients with and without a new diagnosis or treatment, Pearson’s Chi-Square test was used. To assess the relationship between age at time of referral or time between consultations, and outcomes of second opinion, logistic regression models were used.

Results were considered statistically significant if p-value was <0.05. Statistical analyses were performed using SPSS software, version 25 (IBM SPSS Statistics for Windows, Version 25.0).

## Results

### Study population

In total, 196 patients were referred for a second opinion between June 2016 and August 2018. Out of these patients, 23 patients did not visit the clinic or visited the clinic of a different medical specialty. Therefore, 173 patients were included in this study.

### Patient characteristics

Mean age was 42.0 (±16.4) years and the majority of patients were female (69%) (Table 1). Of 173 patients, 65% were referred by their own general practitioner, 21% by a locum general practitioner and 14% by a specialist. At time of referral, a diagnosis had been established by previous doctors in only 15% of patients. Median time between last consultation by a previous physician and first consultation with the physician formulating the second opinion was 97 days (interquartile range (IQR): 43–248 days). Most prevalent presenting symptoms were fatigue (34%), abdominal pain (28%), pain (multifocal) (11%), weight loss (6%), edema (5%) and fever (3%). A list of all other chief complaints can be found in S3 Table.

**Table 1:** Baseline characteristics.

Characteristics	Study population, N = 173
**Age, years**	42.0 (±16.4)
**Gender**	
Male	53 (31%)
Female	120 (69%)
**Referring doctor**	
General practitioner	112 (65%)
Locum general practitioner	37 (21%)
Specialist	24 (14%)
**Diagnosis at time of referral**	
Yes	26 (15%)
No	147 (85%)
Time between consultations*, **days**	
Mean (±SD)	253 (±456)
Median (IQR)	97 (43-248)
**Chief complaint**	
Fatigue	59 (34%)
Abdominal pain	48 (28%)
Pain (multifocal)	19 (11%)
Weight loss	10 (6%)
Edema	8 (5%)
Fever	5 (3%)
Other	24 (14%)

Baseline characteristics are presented as mean (± standard deviation) or number (%). Time between consultations is also presented as median with IQR (25 and 75 percentiles).

Abbreviations: SD = standard deviation, IQR = interquartile range.

* Time between consultations was defined as the number of days between the last consultation with a previous physician and the first consultation with the physician formulating the second opinion.

### Diagnosis

Out of 173 patients, the diagnostic process was completed in 150 patients (87%). In 23 patients (13%) the diagnostic process was still ongoing (4%) or they were lost to follow-up before the diagnostic process was completed (9%). At the conclusion of the second opinion, a diagnosis was established in 38 of all patients (22%) (Table 2). In 23 of these patients (13% of total population) the established diagnosis was considered a new diagnosis. Specified for patients with and without a diagnosis at time of referral, a diagnosis was established in 17 out of 26 patients (65%) with a diagnosis at baseline, including 2 new diagnoses (8%), and 21 out of 147 patients (14%) without a diagnosis at baseline. Most frequently established new diagnoses were Anterior Cutaneous Nerve Entrapment Syndrome (ACNES) (4 patients) and Irritable Bowel Syndrome (IBS) (3 patients). A complete list of new diagnoses established during second opinion is presented in S4 Table. Diagnoses of patients with a diagnosis at time of referral and their diagnosis after second opinion are summarized in S5 Table.

**Table 2:** Diagnoses established during second opinions.

Outcome measure	N	New diagnosis*, N (%)
**Diagnosis established**		
**Total population (N = 173)**	**38 (22%)**	**23 (13%)**
Complete cases (N = 150)	38 (25%)	23 (15%)
Diagnosis established in patients with a diagnosis at time of referral		
All (N = 26)	17 (65%)	2 (8%)
Complete cases (N = 23)	17 (74%)	2 (9%)
Diagnosis established in patients without a diagnosis at time of referral		
All (N = 147)		21 (14%)
Complete cases (N = 127)		21 (17%)
Additional diagnosis established (number of patients)		
Total population (N = 173)	55 (32%)	
Relevant additional diagnosis	50 (29%)	

Data are presented as number of patients (% of patients in category).

* Diagnosis established during second opinion (by the internist formulating the second opinion or during inter-collegial consultation) different from diagnosis at time of referral, or established in a patient without a diagnosis at time of referral.

#### Additional diagnoses

Furthermore, additional diagnoses were established in 55 patients (32%) (Table 2). In 91% of those patients (29% of total population), established additional diagnoses were considered relevant, as treatment for the condition was initiated. Most prevalent additional diagnoses were vitamin (B11, B12, D) and iron deficiencies, urinary tract infection, hypertension and dyslipidemia. A list of all additional diagnoses established during second opinion and their prevalence, can be found in S6 Table.

#### Inter-collegial consultation

During second opinion, 62 patients (36% of total population) were referred for inter-collegial consultation, leading to a total number of 92 consultations. Of the 23 new diagnoses established during second opinion, 6 diagnoses were established during inter-collegial consultation. An overview of inter-collegial consultations during second opinions is presented in S7 Table.

### Treatment

A new treatment was initiated or a change was made in a pre-existing management plan in 97 patients (56%) (Table 3). New treatment mainly involved the prescription of new medication (56%) or the supplementation of vitamins or iron (28%). In 6% of patients receiving new treatment, a change in medication (change in dose or discontinuation) was made. Of 97 patients, 7% received analgesia, 5% received physical therapy, a diet was prescribed in 5%, 4% underwent surgery and 3% received cognitive behavioral therapy.

**Table 3:** Treatment initiated during second opinions.

	N	% of total population
**Treatment initiated**		
Number of treatments	116	-
Number of patients	97	56%
**Treatment type**		
Medication (new)	54 (56%)	31%
Change in medication	6 (6%)	3%
Supplementation*	27 (28%)	16%
Analgesia	7 (7%)	4%
Physical therapy	5 (5%)	3%
Diet	5 (5%)	3%
Surgery	4 (4%)	2%
CBT	3 (3%)	2%
Other^	5 (5%)	3%
**Treatment effects**^+^		
Resolution	6 (6%)	4%
Improvement	27 (28%)	16%
Unchanged	37 (38%)	21%
Worsened	2 (2%)	1%
Unknown	25 (26%)	15%

Data are presented as number (% of patients receiving treatment) and % of total population (N = 173). A patient can receive multiple treatments.

Abbreviations: CBT = Cognitive Behavioral Therapy.

* This includes supplementation of vitamin B11/B12/D and/or iron.

^^^ Other types of treatment included: cyst drainage (N = 1), enteral tube feeding (N = 1), chemoradiation therapy (N = 1), avoidance of sternal pressure (N = 1) and fecal transplantation (N = 1).

^+^ Treatment effects were determined per patient. If a patient received multiple treatments, the overall effect of the treatments combined was used for this analysis.

#### Treatment effects

Regarding treatment effects, resolution of the chief complaint was observed in 6% of patients receiving new treatment and improvement in 28%. Chief complaint had remained unchanged in 38% of patients with a newly initiated treatment, and worsened in 2%. In 26% of patients, treatment effects were unknown as follow-up of the symptoms attributable to the chief complaint were not documented, or patients were discharged or lost to follow-up shortly after the treatment was initiated. Considering the total study population, 19% of all patients received an effective treatment (resolution or improvement of chief complaint) during second opinion.

#### Diagnosis and treatment

Treatment was initiated significantly more frequently in patients with a new diagnosis, established during second opinion (91% vs 51%, p < 0.001). Also, patients with a new diagnosis more frequently received an effective treatment (52% vs 14%, p < 0.001).

### Patient-reported symptomatology

Overall, the chief complaint improved or resolved in 28% of all patients referred for a second opinion (Table 4). Resolution or improvement of the chief complaint was more frequently observed in patients who received a new treatment compared to patients who did not (34% vs 21%), although this difference was not statistically significant (p = 0.06). Patients with a new diagnosis more frequently reported improvement or resolution of symptoms (52% vs 25%, p = 0.006). Patients with neither a new diagnosis nor a new treatment, still reported improved symptomatology in 22% of cases.

**Table 4:** Patient-reported outcome of chief complaint after second opinion.

	**All patients (N = 173)**	
Resolution	9 (5%)	
Improvement	40 (23%)	
Unchanged	56 (32%)	
Worsened	9 (5%)	
Unknown	59 (34%)	
	**With new treatment (N = 97)**	**Without new treatment (N = 76)**
Resolution	6 (6%)	3 (4%)
Improvement	27 (28%)	13 (17%)
Unchanged	37 (38%)	19 (25%)
Worsened	2 (2%)	7 (9%)
Unknown	25 (26%)	34 (45%)
	With new diagnosis* **(N = 23)**	**Without new diagnosis (N = 150)**
Resolution	3 (13%)	6 (4%)
Improvement	9 (39%)	31 (21%)
Unchanged	3 (13%)	53 (35%)
Worsened	1 (4%)	8 (5%)
Unknown	7 (30%)	52 (35%)

Data are presented as N (% of patients in group), for all patients and for patients with or without a new treatment or diagnosis.

* Diagnosis established during second opinion (by the internist formulating the second opinion or during inter-collegial consultation) different from diagnosis at time of referral, or established in a patient without a diagnosis at time of referral.

### Investigations

#### Blood testing

Conventional blood testing was performed in 86% of all patients (Table 5). In 89% of these cases, conventional blood testing had already been carried out by the previous physician, but was repeated during second opinion. Conventional blood testing led to relevant information in 23% of all cases in which conventional blood testing was performed: 23% of cases in which conventional blood testing was repeated, and 24% of cases in which conventional blood testing was performed for the first time. It showed anomalous results contributing to the establishment of a diagnosis in 4% of all cases. Additional blood tests were performed in 72% of all patients, leading to relevant information in 17% and leading to anomalous results contributing to the establishment of a diagnosis in 2% of these patients.

**Table 5:** Performed investigations during second opinions.

Investigation	Performed	Repeated*	Relevant information^			Anomalous result contributing to diagnosis^+^
		% of performed	% of performed	% of new	% of repeated	% of performed
**Blood tests**						
Conventional^#^	148 (86%)	89%	23%	24%	23%	4%
Additional^&^	124 (72%)	2%	17%	17%	-	2%
**Urinalysis**	92 (53%)	42%	14%	13%	15%	1%
**Microbiology**	**87 (50%)**					
Viral	69 (40%)	6%	6%	5%	25%	-
Bacterial	72 (42%)	8%	17%	15%	33%	-
Other	25 (15%)	4%	20%	21%	-	-
**Radiology**	**84 (49%)**					
X-ray	55 (32%)	33%	7%	11%	-	-
Sonography	30 (17%)	23%	23%	22%	29%	10%
CT	32 (19%)	31%	28%	27%	30%	3%
MRI	8 (5%)	-	75%	75%	-	-
PET-CT/SPECT	8 (5%)	25%	75%	67%	100%	38%
**Endoscopy**	16 (9%)	31%	25%	27%	20%	6%
**Pathology**	25 (15%)	28%	32%	33%	29%	24%

Data are presented as number of patients (% of total population) with % of performed investigations that were repeated, led to relevant information and contributed to the diagnosis. Relevant information rates are also specified for new and repeated investigations.

* Investigation performed during second opinion, which had already been performed by a previous physician before the start of the second opinion. Reassessments of results, images or tissue obtained by previous investigations and transferred from a previous hospital to the UMC Utrecht, were not considered investigations as the actual investigations were not performed during second opinion, so reassessments were also not considered repeated investigations.

^^^ Information not known from previous investigations leading to either the establishment of a diagnosis or additional diagnosis, the initiation of a new treatment or the requirement for another investigation for further assessment.

^+^ Anomalous results discovered by an investigation performed during second opinion, contributing to the establishment of a diagnosis.

^#^ Blood tests regularly performed during second opinions (specified in S2 Table).

^&^ Blood tests not included in conventional blood testing.

#### Urinalysis and microbiology

Urinalysis was carried out in 53% of patients (repetition rate = 42%). Relevant information was discovered in 14% of these patients (13% of new investigations, 15% of repeated investigations). Anomalous results discovered by urinalysis contributed to the establishment of a diagnosis in only 1% of patients it was performed in. In 50% of all patients, microbiology tests were performed, mostly focused on bacterial and viral pathogens. Repetition rates were low (4-6%). Overall relevant information rates ranged from 6% (viral) to 20% (other: parasites, fungi, protozoa). Noticeably, anomalous results of microbiology tests did not once contribute to the establishment of a diagnosis.

#### Radiology

Radiological tests were performed in 49% of all patients. X-ray was most frequently performed (32%), MRI and PET-CT/SPECT were each only performed in 5% of patients. Repetition rates ranged from 23% to 33%, except for MRI, which was never a repetition of a previously conducted investigation. When repeated, sonography and CT lead to relevant information in 29% and 30% of cases respectively, while repeated PET-CT’s or SPECT’s always, and repeated X-rays never lead to relevant new information. Regarding new investigations, relevant information rates were: 11% for X-ray, 22% for sonography, 27% for CT, 75% for MRI and 67% for PET-CT/SPECT. When all performed tests, repeated or new, were considered, relevant information rates were high for MRI and PET-CT/SPECT (75%), intermediate for sonography and CT (23% and 28%), and low for X-ray (7%). When PET-CT or SPECT was performed, it led to the discovery of anomalous results relevant to the diagnosis in 38% of patients. Anomalous results of sonography and CT led to a diagnosis in 10% and 3% of cases respectively. X-ray never showed anomalous results contributing to the establishment of a diagnosis.

*Endoscopy and pathology*. Endoscopic procedures were carried out in 9% of patients (repetition rate = 31%), leading to the discovery of relevant information in 25% (27% of new investigations, 20% of repeated investigations), and to anomalous results contributing to the establishment of a diagnosis in 6% of these patients. In 15% of all patients, pathology tests were performed (repetition rate = 28%), leading to relevant information in 32% (33% of new investigations, 29% of repeated investigations), and anomalous results contributing to the diagnosis in 24% of patients.

### Follow-up

Median time to diagnosis was 64 days (IQR (interquartile range): 25–128 days), for patients in whom a diagnosis was established (Table 6). When regarding new diagnoses only, median time to diagnosis was 68 days (IQR: 35–153 days). Median time to discharge was 75 days (IQR: 31–144 days) for patients whose second opinions were completed. Median time spent at the internal medicine outpatient clinic was 70 minutes (IQR: 60–90 minutes), and median time spent at any outpatient clinic of the UMC Utrecht (in the context of the second opinion) was 80 minutes (IQR: 60–135 minutes).

**Table 6:** Time spent during second opinions.

Measure	Mean (±SD)	Median	IQR (25 – 75 percentiles)	Total
**Time to diagnosis, days**				
All* (N = 38)	96 (±130)	64	25 - 128	
New^ (N = 23)	117 (±153)	68	35 - 153	
**Time to discharge, days**				
Complete cases (N = 143)	109 (±108)	75	31 - 144	
Not yet discharged^+^ (N = 14)	499 (±193)	433	335 - 619	
Lost to follow-up^#^ (N = 16)	99 (±94)	49	20 - 185	
**Time in clinic, minutes**				
Internal medicine	80 (±31)	70	60 - 90	230 hours
All outpatient clinics	114 (±93)	80	60 - 135	330 hours

Data are presented as mean (±SD), median and IQR (25 – 75 percentiles).

Abbreviations: IQR = interquartile range.

* All diagnoses established by the doctor formulating the second opinion plus relevant diagnoses established during consultation by another specialist.

^^^ Diagnosis established during second opinion (by the internist formulating the second opinion or during inter-collegial consultation) different from diagnosis at time of referral, or established in a patient without a diagnosis at time of referral.

^+^ Patients not yet discharged at the start of the study, 1 May 2019 was used as time of discharge.

^#^ Patients lost to follow-up, last visit was used as time of discharge.

### Determinants of outcome

Statistically significant differences in outcome between patient groups based on gender, referring doctor, chief complaint or presence of a diagnosis at time of referral, were not found (Table 7). Neither was there a significant relationship between age or time between consultations, and chance of a new diagnosis or a new (effective) treatment (Table 8). However, when specifically comparing patients with abdominal pain to patients with fatigue, a new diagnosis was more frequently established in patients with abdominal pain (23% vs 9%, p = 0.037).

**Table 7:** Potential determinants of outcomes of second opinions.

Determinant	New diagnosis (N = 23)	p-value	New treatment (N = 97)	p-value	Effective treatment* (N = 33)	p-value
**Gender**						
Male (N = 53)	5 (9%)	0.320	29 (55%)	0.812	8 (15%)	0.376
Female (N = 120)	18 (15%)		68 (57%)		25 (21%)	
**Referring doctor**						
General practitioner (N = 112)	16 (14%)	0.858	59 (53%)	0.409	20 (18%)	0.389
Locum general practitioner (N = 37)	4 (11%)		22 (60%)		6 (16%)	
Specialist (N = 24)	3 (13%)		16 (67%)		7 (29%)	
**Chief complaint**^						
Fatigue (N = 59)	5 (9%)	0.108	31 (53%)	0.632	9 (15%)	0.715
Abdominal pain (N = 48)	11 (23%)		30 (63%)		10 (21%)	
Pain (multifocal) (N = 19)	3 (16%)		9 (47%)		3 (16%)	
Other (N = 47)	4 (9%)		27 (57%)		11 (23%)	
**Diagnosis at time of referral**						
Yes (N = 26)	2 (8%)	0.361	15 (58%)	0.856	5 (19%)	0.983
No (N = 147)	21 (14%)		82 (56%)		28 (19%)	

Data are presented as number (% of subcategory). P-values for differences in outcome within categories are given.

* Initiated treatment leading to improvement or resolution of the chief complaint.

^^^ Chief complaints with a prevalence of ≥ 10% were used as separate groups, remaining chief complaints were placed in ‘Other’.

**Table 8:** Other potential determinants of outcomes.

	New diagnosis			New treatment			Effective treatment		
	Yes (N = 23)	No (N = 150)	p-value*	Yes (N = 97)	No (N = 76)	p-value*	Yes (N = 33)	No (N = 140)	p-value*
**Age, mean (±SD)**	42.1 (±17.5)	42.0 (±16.2)	0.960	41.9 (±15.6)	42.1 (±17.4)	0.931	41.0 (±16.5)	42.2 (±16.4)	0.722
**Time between consultations, mean (±SD)**	226 (±339)	258 (±472)	0.765	256 (±481)	251 (±425)	0.946	267 (±564)	250 (±428)	0.852

Data are presented as mean (± standard deviation).

* p-values from logistic regression models for the relationships between age and new diagnosis/(effective) treatment, and time between consultations and new diagnosis/(effective) treatment.

## Discussion

During second opinions in a general internal medicine outpatient clinic of an academic hospital, a new diagnosis was established in 13% of patients, while overall, resolution or improvement of the chief complaint was achieved in 28% of patients. In approximately one third of patients a relevant additional diagnosis was established, and in over half of all patients, a new treatment was initiated. Treatment, whether a new diagnosis was established or not, led to improvement or resolution of the chief complaint in 34% of patients. Many investigations were carried out, often repeating previously performed investigations. Anomalous results from investigations rarely contributed to the establishment of a diagnosis.

Regarding the establishment of a new diagnosis, results presented in this study are very similar to findings of the two previous studies exploring the outcomes of second opinions in internal medicine (both in Dutch academic hospitals), with a new diagnosis being established in approximately 10% of patients in these studies [7, 8]. We add to the body of evidence by detailing the diagnostic process and outcome. When compared to second opinions in other medical specialties, diagnostic value of second opinions in internal medicine is low. Studies in other medical specialties have shown that a new diagnosis is established in approximately 30-60% of patients, ranging from 30% in surgical oncology to 60% in orthopedic surgery [10, 12, 13, 18, 20, 23, 24]. This difference might be explained by the fact that, in this study and in previous studies [7, 8], up to 85% of patients referred for a second opinion in internal medicine, had poorly defined conditions without a diagnosis at the time of referral. This usually concerns patients with a high suspicion of medically unexplained physical symptoms, in whom a diagnosis cannot be easily established. Also, part of the diagnoses that were established in this study, are diagnoses without objective criteria and for which treatment options are lacking. One could question the value of the establishment of these kinds of diagnoses. The same applies to additional diagnoses, such as iron and vitamin deficiencies, which were frequently established in this study.

In this study, a new diagnosis was more frequently established in patients presenting with abdominal pain when compared to patients presenting with fatigue. This was also observed in small numbers of patients presenting with abdominal pain or fatigue in a previous study [7], although this finding is not consistent [8]. The fact that more new diagnoses were established in patients with abdominal pain in our study is most likely related to the fact that ACNES was the most frequently established new diagnosis (S4 Table). ACNES is known to be a poorly recognized and commonly underdiagnosed cause of abdominal pain [33–36]. Therefore, it is likely that ACNES is sometimes not recognized by the original physician, but the diagnosis is established by the physician formulating the second opinion, as internists in our center are aware of the fact that ACNES is a commonly underdiagnosed cause of abdominal pain. This likely contributed to the higher number of new diagnoses established in patients presenting with abdominal pain.

This is the first study exploring treatment initiation and patient-reported symptomatology during second opinions in internal medicine to date. Noticeably, this study showed that the proportion of patients who received a new treatment was substantially larger than the proportion of patients in whom a new diagnosis was established. This indicates that, even though a new diagnosis was strongly related to the initiation of a new treatment in this study, a new treatment is also frequently initiated in patients in whom no diagnosis was established. In addition, resolution or improvement of symptoms was also frequently achieved in patients who did not receive a new treatment, nor a new diagnosis. This means that the yield of second opinions is not limited to the establishment of diagnoses. However, it is commonly known that a placebo effect can play a substantial role in patient-reported symptomatology and treatment effects. In the absence of a control group, it is hard to determine what part of the treatment effects and reported improved symptoms in this study, are attributable to a placebo effect. It is likely that information and reassurance provided by the physician carrying out the second opinion, can lead to improved symptoms, as it was shown to increase patient satisfaction in a previous study [7]. One could argue that this is part of the value of second opinions, whether it is based on a placebo effect or not.

This is the first study that thoroughly analyzed investigations performed in the context of second opinions. One previous study in an internal medicine outpatient clinic reported that, depending on the type of investigation (for example blood testing, urinalysis or radiology), approximately 40-90% of investigations performed by original physicians were repeated by the physician formulating the second opinion [8]. Our study focused on how many of the performed investigations during second opinion were in fact a repetition of investigations already carried out by the original physicians. Repetition rates for radiological tests seemed to be lower in our study, possibly caused by the fact that, nowadays, information from case records is more easily transferred between hospitals. Also, a radiological second opinion of investigations performed in other hospitals, can be easily obtained. Repetition of investigations could be seen as waste. However, noticeably, in this study, repeated investigations led to the discovery of relevant information relatively frequently, which is in contrast with the aforementioned study [8]. Partly, this is due to the fact that in a considerable number of patients conventional blood testing was considered repeated, while in fact a small share of the tests had not been performed before. Conventional tests that had not been performed before mostly included vitamin and iron tests, which often led to relevant information. Thus, conventional blood testing often showed relevant information when repeated, but relevant information was often only found in the share of tests that were actually not a repetition. The relatively high relevant information rates for repeated microbiology, radiology, endoscopy and pathology tests are remarkable. These results suggest that, when doctors formulating second opinions believe it is necessary, repeating investigations can be useful. Time lapsed between original and repeated investigations was not assessed in this study, so a statement on the possible relationship between amount of time between investigations and relevance of results cannot be made.

One of the strengths of this study is the fact that it is the most extensive research on second opinions in general internal medicine to date. Additional diagnoses, treatment effects, patient-reported symptomatology, relevance of all performed investigations and follow-up time had never been assessed before. Also, this is the first study exploring the value of second opinions in internal medicine in ten years’ time. Finally, strengths of this study include the large population size and the fact that all patients who visited our clinic for a second opinion in the given time frame were included in the study, so selection bias was avoided.

A limitation of this study is the retrospective design and the fact that all outcomes were based on case records. Nevertheless, most important outcome measures, such as diagnoses and treatment, generally are carefully documented by physicians in case records, including correspondence, so a considerable impact of the study design on end points is unlikely. However, treatment effects were not always accurately documented, and one could question whether improvement of symptoms after the initiation of treatment is always caused by the treatment. It is likely that in some patients, symptoms resolve due to a placebo effect. So, treatment effects might be overestimated in this study. Furthermore, the fact that in some patients diagnostic process was incomplete, could be seen as a limitation of this study. However, by reporting our outcome (new diagnosis) as percentage of the total of referred patients, we describe current practice and thus approximate the real benefit of referral for second opinion in general internal medicine. Finally, there was a limitation in the way relevance of information discovered by investigations was determined. Information was only considered relevant in case of anomalous results leading to the establishment of a diagnosis or additional diagnosis, the initiation of treatment, or the requirement for another investigation for further assessment, while normal results or negative tests might be relevant in establishing or ruling out a diagnosis as well.

## Conclusion

In conclusion, this extensive research on the outcomes of second opinions in general internal medicine, has shown that a new diagnosis is established in 13% of patients. Patients in whom a new diagnosis is established benefit more from second opinions, but the value of second opinions may not be limited to the establishment of diagnoses, as patients without a new diagnosis also frequently receive treatment and report improvement of symptoms. Overall, at least 28% of patients benefit from second opinions, as resolution or improvement of symptoms is achieved. Whether this is because of the consultation, diagnosis, treatment or the natural course of the complaint or disease could not be ascertained. Remarkably, a large number of investigations are performed and repeated during second opinions, while these investigations rarely contribute to the establishment of a diagnosis. Despite of that, this study has shown that second opinions in internal medicine are valuable in terms of the establishment of diagnoses, initiation of treatment and improvement of symptoms, in a considerable number of patients.

## Supporting information

S1 Table Glossary of terms used throughout the manuscript. (DOCX)

S2 Table List of all blood tests regarded as conventional blood tests. (DOCX)

S3 Table Other chief complaints. (DOCX)

S4 Table New diagnoses established during second opinions. (DOCX)

S5 Table Diagnosis after second opinion in patients with a diagnosis at time of referral. (DOCX)

S6 Table Additional diagnoses established during second opinions. (DOCX)

S7 Table Inter-collegial consultation during second opinions. (DOCX)

S1 File Minimal data set. (XLSX)
