# Supplementary material for: Outcomes of second opinions in general internal medicine
Source: PLoS One. 2020 Jul 9;15(7):e0236048. doi: 10.1371/journal.pone.0236048 (PMC7347190; doi:10.1371/journal.pone.0236048)
Supplement: S1 Table — (DOCX) [file pone.0236048.s001.docx]

| **S1 Table. Glossary of terms used throughout the manuscript.** | |
| --- | --- |
| **Term** | **Definition** |
| Referring doctor | The doctor who referred the patient for the second opinion and wrote the referral letter. |
| Time between consultations | Number of days between the last consultation with a previous physician and the first consultation with the physician formulating the second opinion. |
| Chief complaint | Main complaint mentioned by the patient during the first visit to the clinic. |
| Diagnosis at time of referral | Diagnosis established by referring doctor or another physician before second opinion, as documented by the referring doctor in the referral letter. |
| Diagnosis by doctor formulating the second opinion | Diagnosis established by the internist carrying out the second opinion. |
| Diagnosis established during inter-collegial consultation | Diagnosis established by a doctor from another medical specialty during inter-collegial consultation requested by the internist formulating the second opinion. |
| New diagnosis | Diagnosis established during second opinion (by the internist formulating the second opinion or during inter-collegial consultation) different from diagnosis at time of referral, or established in a patient without a diagnosis at time of referral. |
| Additional diagnosis | Diagnosis established during second opinion, concerning a condition which cannot cause the chief complaint. |
| Relevant additional diagnosis | Additional diagnosis leading to the initiation of treatment for that condition. |
| New treatment | The initiation of (a change in) medication, vitamin/iron supplementation, analgesia, therapy, dietary prescriptions or surgery, during second opinion. |
| Treatment effects | Effects of new treatment initiated during second opinion, on the chief complaint as reported by the patient and documented by the doctor in the case record. |
| Effective treatment | New treatment leading to the resolution or improvement of the chief complaint. |
| Patient-reported symptomatology | Outcome of chief complaint at the end of second opinion as reported by the patient and documented by the doctor in the case record. |
| Conventional blood testing | Blood tests regularly performed during second opinions (specified in S2 Table). |
| Additional blood tests | Blood tests not included in conventional blood testing. |
| New investigation | An investigation performed during second opinion, which had not been performed by a previous physician. |
| Repeated investigation | An investigation was considered a repeated investigation if the investigation had already been performed by a previous physician before the start of the second opinion, and the exact same investigation was then performed again during the second opinion. Reassessments of results, images or tissue were not considered investigations or repeated investigations. |
| Relevant information | Information not known from previous investigations leading to either the establishment of a diagnosis or additional diagnosis, the initiation of a new treatment or the requirement for another investigation for further assessment. |
| Overall relevant information rate | Percentage of investigations that lead to relevant information, for new and repeated investigations combined. |
| Anomalous results contributing to diagnosis | Anomalous results discovered by an investigation performed during second opinion, contributing to the establishment of a diagnosis. |
| Time to diagnosis | Number of days between the first visit to the clinic and the moment the diagnosis was established and discussed with the patient, as documented in the case record. |
| Time to discharge | Number of days between the first visit to the clinic and the last visit to the clinic, or other departments of the hospital, as part of the diagnostic process or treatment of the chief complaint. |
| Time spent in the clinic | Total amount of time (in minutes) reserved for the patients’ appointments at the internal medicine outpatient clinic, as well as for appointments by phone. |
| Total time spent in the clinic | Total amount of time (in minutes) reserved for the patients’ appointments, regarding the chief complaint, at any outpatient clinic in the UMC Utrecht, as well as for appointments by phone. |
