# Supplementary material for: Outcomes of second opinions in general internal medicine
Source: PLoS One. 2020 Jul 9;15(7):e0236048. doi: 10.1371/journal.pone.0236048 (PMC7347190; doi:10.1371/journal.pone.0236048)
Supplement: S2 Table — (DOCX) [file pone.0236048.s002.docx]

| S2 Table. List of all blood tests regarded as conventional blood tests. | | |
| --- | --- | --- |
| Blood chemistry | **Hematology** | **Endocrinology** |
| Natrium | Hemoglobin | Thyroid-stimulating hormone |
| Kalium | Hematocrit | Free thyroxine (free T4) |
| Calcium | Erythrocytes | 25-hydroxyvitamin D |
| Phosphate | Mean corpuscular volume (MCV) |  |
| Uric acid | Mean corpuscular hemoglobin (MCH) |  |
| Creatinine | Mean corpuscular hemoglobin concentration (MCHC) |  |
| Estimated Glomerular Filtration Rate (eGFR) | Thrombocytes |  |
| Bilirubin | Leukocytes |  |
| Alkaline phosphatase | Neutrophilic granulocytes |  |
| Gamma glutamyltransferase (GGT) | Basophilic granulocytes |  |
| Aspartate transaminase (AST) | Eosinophilic granulocytes |  |
| Alanine transaminase (ALT) | Lymphocytes |  |
| Lactate dehydrogenase (LD) | Monocytes |  |
| Creatine kinase (CK) | Erythrocyte sedimentation rate (ESR) |  |
| Amylase |  |  |
| Lipase |  |  |
| Albumin |  |  |
| C-reactive protein (CRP) |  |  |
| Cholesterol |  |  |
| Triglycerides |  |  |
| High-density lipoprotein (HDL) |  |  |
| Low-density lipoprotein (LDL) |  |  |
| Ferritin |  |  |
| Transferrin |  |  |
| Transferrin iron saturation |  |  |
| Iron |  |  |
| Folic acid (vitamin B11) |  |  |
| Vitamin B12 |  |  |
|  |  |  |
| Glucose |  |  |
