# Supplementary material for: Outcomes of second opinions in general internal medicine
Source: PLoS One. 2020 Jul 9;15(7):e0236048. doi: 10.1371/journal.pone.0236048 (PMC7347190; doi:10.1371/journal.pone.0236048)
Supplement: S3 Table — (DOCX) [file pone.0236048.s003.docx]

| S3 Table. Other chief complaints. | |
| --- | --- |
| Chief complaint | **Prevalence (Total: N = 24)** |
| Headache | 3 |
| Syncope | 3 |
| Ascites | 2 |
| Flushes | 2 |
| Joint complaints | 2 |
| Pruritus | 2 |
| Anaphylaxis | 1 |
| Diarrhea | 1 |
| Dyspnea | 1 |
| Hiccups | 1 |
| Hyperhidrosis | 1 |
| Hypothermia | 1 |
| Nausea | 1 |
| Skin bumps | 1 |
| Weight gain | 1 |
| Wounds | 1 |
| Prevalence of chief complaints is reported as number. | |
