# Supplementary material for: Outcomes of second opinions in general internal medicine
Source: PLoS One. 2020 Jul 9;15(7):e0236048. doi: 10.1371/journal.pone.0236048 (PMC7347190; doi:10.1371/journal.pone.0236048)
Supplement: S4 Table — (DOCX) [file pone.0236048.s004.docx]

| S4 Table. New diagnoses established during second opinions. | |
| --- | --- |
| New diagnosis* | **Prevalence (Total: N = 23)** |
| Anterior Cutaneous Nerve Entrapment Syndrome (ACNES) | 4 |
| Irritable Bowel Syndrome (IBS) | 3 |
| Abdominal angina | 1 |
| Chronic Fatigue Syndrome (CFS) | 1 |
| Erythromelalgia | 1 |
| Facet syndrome | 1 |
| Familial Mediterranean Fever (FMF) | 1 |
| Fibromyalgia | 1 |
| Hyperventilation syndrome | 1 |
| Iron deficiency | 1 |
| Metastatic mammary carcinoma | 1 |
| Morbus Castleman | 1 |
| Morbus Crohn | 1 |
| Non-alcoholic steatohepatitis (NASH) | 1 |
| Postural Orthostatic Tachycardia Syndrome (POTS) | 1 |
| Schnitzler syndrome | 1 |
| Supragastric belching | 1 |
| Tietze syndrome | 1 |
| Prevalence of diagnoses is presented as number. Diagnoses established (partly) based on objectifiable findings from biochemical, radiological or pathological examinations are in bold.  * Diagnosis established during second opinion (by the internist formulating the second opinion or during inter-collegial consultation) different from diagnosis at time of referral, or established in a patient without a diagnosis at time of referral. | |
