# Supplementary material for: Outcomes of second opinions in general internal medicine
Source: PLoS One. 2020 Jul 9;15(7):e0236048. doi: 10.1371/journal.pone.0236048 (PMC7347190; doi:10.1371/journal.pone.0236048)
Supplement: S5 Table — (DOCX) [file pone.0236048.s005.docx]

| S5 Table. Diagnosis after second opinion in patients with a diagnosis at time of referral. | |
| --- | --- |
| Diagnosis at time of referral (N = 26) | **Diagnosis after second opinion (N = 17)** |
| Anterior Cutaneous Nerve Entrapment Syndrome (ACNES) | Anterior Cutaneous Nerve Entrapment Syndrome (ACNES) |
| Chronic Fatigue Syndrome (CFS) | Chronic Fatigue Syndrome (CFS) |
| Chronic idiopathic urticaria | Chronic idiopathic urticaria |
| Erythema Nodosum | Erythema Nodosum |
| Fibromyalgia | Fibromyalgia |
| Fibromyalgia | Fibromyalgia |
| Fibromyalgia | - |
| Fibromyalgia + Chronic Obstructive Pulmonary Disease (COPD) + Hypothyroidism | Fibromyalgia + Chronic Obstructive Pulmonary Disease (COPD) + Hypothyroidism |
| Graves’ disease | - |
| Iron deficiency anemia | Iron deficiency anemia |
| Iron deficiency anemia | Iron deficiency anemia |
| Irritable Bowel Syndrome (IBS) | **Anterior Cutaneous Nerve Entrapment Syndrome (ACNES)** |
| Irritable Bowel Syndrome (IBS) | **Supragastric belching** |
| Irritable Bowel Syndrome (IBS) | Irritable Bowel Syndrome (IBS) |
| Irritable Bowel Syndrome (IBS) | Irritable Bowel Syndrome (IBS) |
| Irritable Bowel Syndrome (IBS) | - |
| Irritable Bowel Syndrome (IBS) | - |
| Irritable Bowel Syndrome (IBS) | - |
| Irritable Bowel Syndrome, post-infectious (IBS) | Irritable Bowel Syndrome, post-infectous (IBS) |
| Leukocytoclastic vasculitis | Leukocytoclastic vasculitis |
| Pain amplification syndrome | Pain amplification syndrome |
| Pancreatic insufficiency | - |
| Splenic cyst | Splenic cyst |
| Urticaria factitia | - |
| Vitamin B12 deficiency anemia | - |
| Yellow nail syndrome | - |
| New diagnoses are in bald. If no diagnosis after second opinion is mentioned, this means that the diagnosis at time of referral was not confirmed and that a new diagnosis was not established. | |
