# Supplementary material for: Outcomes of second opinions in general internal medicine
Source: PLoS One. 2020 Jul 9;15(7):e0236048. doi: 10.1371/journal.pone.0236048 (PMC7347190; doi:10.1371/journal.pone.0236048)
Supplement: S6 Table — (DOCX) [file pone.0236048.s006.docx]

| S6 Table. Additional diagnoses established during second opinions. | |
| --- | --- |
| Additional diagnosis* | **Prevalence**  **(Total: N = 55 patients, N = 78 diagnoses)** |
| Iron deficiency | 11 |
| Vitamin B12 deficiency | 10 |
| Folic acid (vitamin B11) deficiency | 8 |
| Urinary tract infection | 8 |
| Vitamin D deficiency | 7 |
| Hypertension | 5 |
| Dyslipidemia | 4 |
| Adrenal incidentaloma | 1 |
| Angina Pectoris | 1 |
| Baker’s cyst | 1 |
| Breast cyst | 1 |
| Bursitis | 1 |
| Candidiasis | 1 |
| Chronic idiopathic urticaria | 1 |
| Erythema chronicum migrans | 1 |
| Focal nodular hyperplasia of the liver | 1 |
| Gallbladder polyp | 1 |
| Gastric fundic gland polyp | 1 |
| IgG2 subclass deficiency | 1 |
| IgM Monoclonal Gammopathy of Unknown Significance (MGUS) | 1 |
| Kidney cyst | 1 |
| Mannose-binding lectin deficiency | 1 |
| Obstructive Sleep Apnea Syndrome (OSAS) | 1 |
| Panniculitis mesenterica | 1 |
| Parasitic gastroenteritis | 1 |
| Peptic duodenitis | 1 |
| Pulmonary embolism | 1 |
| Pulmonary emphysema | 1 |
| Sjögren Syndrome | 1 |
| Specific antibody deficiency | 1 |
| Syndrome of inappropriate antidiuretic hormone secretion (SIADH) | 1 |
| Tubular adenoma | 1 |
| Prevalence of additional diagnoses is presented as number. A patient may have multiple additional diagnoses.  * Diagnosis established during second opinion, concerning a condition which cannot cause the chief complaint. | |
