# Supplementary material for: Outcomes of second opinions in general internal medicine
Source: PLoS One. 2020 Jul 9;15(7):e0236048. doi: 10.1371/journal.pone.0236048 (PMC7347190; doi:10.1371/journal.pone.0236048)
Supplement: S7 Table — (DOCX) [file pone.0236048.s007.docx]

| S7 Table. Inter-collegial consultation during second opinions. | | | |
| --- | --- | --- | --- |
|  | **N** | **% of referred patients**  **(N = 62)** | **% of total population**  **(N =173)** |
| Patients referred for consultation  Total number of consultations | 62  92 |  | 36%  - |
| Specialties  Gastroenterology  Rheumatology  Dermatology  Neurology  Cardiology  General surgery  Hematology  Anesthesiology  Gynecology  Otorhinolaryngology  Pulmonology  Urology  Other* | 20  11  10  9  8  6  6  4  4  3  3  3  5 | 32%  18%  16%  15%  13%  10%  10%  7%  7%  5%  5%  5%  8% | 12%  6%  6%  5%  5%  3%  3%  2%  2%  2%  2%  2%  3% |
| Diagnosis during consultation  Diagnosis chief complaint  Additional diagnosis | 6  4 | 10%  6% | 3%  2% |
| Patients and consultations are presented as number (% of patients in category), with % of total population (N = 173). A patient may be referred to multiple specialties.  * Other specialties included: ophthalmology (N = 2), cardiothoracic surgery (N = 1), orthopedic surgery (N = 1) and oncology (N = 1). | | | |
